# Impact of MRI slice thickness and fiducial marker orientation on geometric accuracy in multi‐echo GRE imaging for prostate radiotherapy planning

**DOI:** 10.1002/acm2.70741

**Published:** 2026-08-13

**Authors:** Aika Hayashi, Yasukata Takahashi, Naoko Sanuki, Yasufumi Yamashita, Yoshitaka Suzuki, Kanae Matsuura, Hiroe Muto

**Affiliations:** ^1^ Graduate School of Medical Science Suzuka University of Medical Science Suzuka Mie Japan; ^2^ Department of Central Radiology Yokkaichi Municipal Hospital Yokkaichi Mie Japan; ^3^ Department of Radiology Keio University School of Medicine Shinjuku‐ku Tokyo Japan; ^4^ Department of Radiology Yokkaichi Municipal Hospital Yokkaichi Mie Japan; ^5^ Department of Radiology Mie University Hospital Tsu Mie Japan; ^6^ Department of Radiological Technology, Faculty of Health Science Suzuka University of Medical Science Suzuka Mie Japan; ^7^ Center for Fundamental Education of Health Professions Suzuka University of Medical Science Suzuka‐city Mie Japan

**Keywords:** Gold fiducial markers, Image registration, Magnetic resonance imaging, Prostate cancer

## Abstract

**Background:**

Precise target definition in prostate radiotherapy requires accurate computed tomography (CT)‐magnetic resonance imaging (MRI) image registration using gold fiducial markers (GFMs). While multi‐echo gradient recalled echo (ME GRE) sequences facilitate GFM identification, the quantitative impact of acquisition parameters on physical measurement accuracy remains insufficiently understood.

**Purpose:**

This study evaluates how MRI slice thickness and marker orientation angle jointly affect GFM visualization and geometric accuracy to characterize the resulting localization uncertainties.

**Methods:**

A kiwifruit‐based phantom (T1: 1603.3 ms; T2: 72.3 ms) that mimics the relaxation times of the human prostate was imaged with 3.0T MRI. Imaging was performed using a 3D ME GRE sequence with three slice thicknesses (1.0, 2.0, and 3.0 mm) and 12 orientation angles ranging from horizontal to 90°. Five multidisciplinary observers independently identified marker coordinates while blinded to the acquisition parameters. Subjective confidence scores were also recorded.

**Results:**

Two‐way analysis of variance (ANOVA) revealed significant main effects for slice thickness (*F*(2, 144) = 32.03, *p* < 0.001) and orientation angle (*F*(11, 144) = 4.06, *p* < 0.001), with significant interaction between them (*F* (22, 144) = 2.72, *p* < 0.001). Mean measurement errors with 95% confidence intervals (CI) were 0.96 mm (95% CI: 0.70–1.22) for 1.0 mm, 1.49 mm (95% CI: 1.23–1.75) for 2.0 mm, and 2.00 mm (95% CI: 1.74–2.26) for 3.0 mm slices. The peak error reached 3.10 mm under the combination of a 3.0 mm slice and 15° orientation. Subjective confidence scores showed a significant negative correlation with physical measurement errors (*r* = −0.296, *p* < 0.001).

**Conclusions:**

MRI slice thickness and marker orientation significantly impact GFM visualization accuracy. The results suggest that a 1.0 mm slice thickness offers a technical advantage for maintaining sub‐millimeter localization accuracy across various marker orientations and highly desirable for high‐precision radiotherapy planning.

## 1 INTRODUCTION

In the workflow for targeting localized prostate cancer for intensity‐modulated radiation therapy (IMRT), it is common to define the treatment target by combining computed tomography (CT) and magnetic resonance imaging (MRI).^1^ The image registration approaches^2^, ^3^ used to combine the images from the two modalities typically utilize techniques such as mutual information^4^, ^5^, ^6^ and gold fiducial markers (GFM).^7^, ^8^ Recently, it has become a common procedure to implant GFM in the prostate of patients undergoing radiation therapy for prostate cancer.^9^ When planning IMRT for the prostate, the acquisition of T2‐weighted MRI images alongside therapeutic planning CT images is considered useful for clear prostate visualization.^10^ However, GFMs placed in the prostate are difficult to visualize on T2‐weighted images.^10^ When visualization of GFMs is necessary, the multi‐echo gradient recall echo (ME GRE) sequence offers advantages because it is sensitive to changes in magnetic susceptibility,^11^ which enables GFM visualization as signal voids—a feature typically absent in T2‐weighted images.^9^ While such visualization performance is critical for marker identification, it does not inherently guarantee geometric accuracy—defined as the degree to which the depicted coordinates and dimensions represent the true physical geometry. ME GRE allows the signal‐to‐noise ratio of T2‐weighted images to be maintained while reducing the image distortion caused by magnetic susceptibility,^12^, ^13^ although the inter‐tissue contrast of ME GRE is inferior to that of T2‐weighted images. Despite the superior visualization performance of ME GRE, previous investigations have predominantly focused on the subjective visibility of markers as signal voids, rather than their geometric fidelity. Reports that quantitatively link acquisition parameters to the physical measurement accuracy of GFMs are limited. This study provides a novel contribution by shifting the focus from subjective detectability to the rigorous quantification of geometric uncertainty. We investigated the joint impact of slice thickness and GFM orientation angle, believing that such clarification provides a necessary physical baseline for understanding localization uncertainties in prostate contouring and position registration accuracy during radiation therapy planning.^14^

## 2 METHODS

### 2.1 Phantom fabrication

To simulate human prostate tissue, a customized phantom was fabricated using a kiwi fruit, which exhibits T1 and T2 relaxation times like those of the human prostate.^15^ The longitudinal (T1) and transverse (T2) relaxation times of the kiwi fruit were measured using a MIXED sequence(a pulse sequence designed for simultaneous T1 and T2 mapping) on the MRI system, and the values were quantified using the vendor‐provided on‐device region‐of‐interest (ROI) analysis software. The measured T1 and T2 values were 1603.3 ms and 72.3 ms, respectively. The kiwi fruit had dimensions of approximately 40.4 mm in height, 52.8 mm in width, and 66.2 mm in length.

**FIGURE 1:**
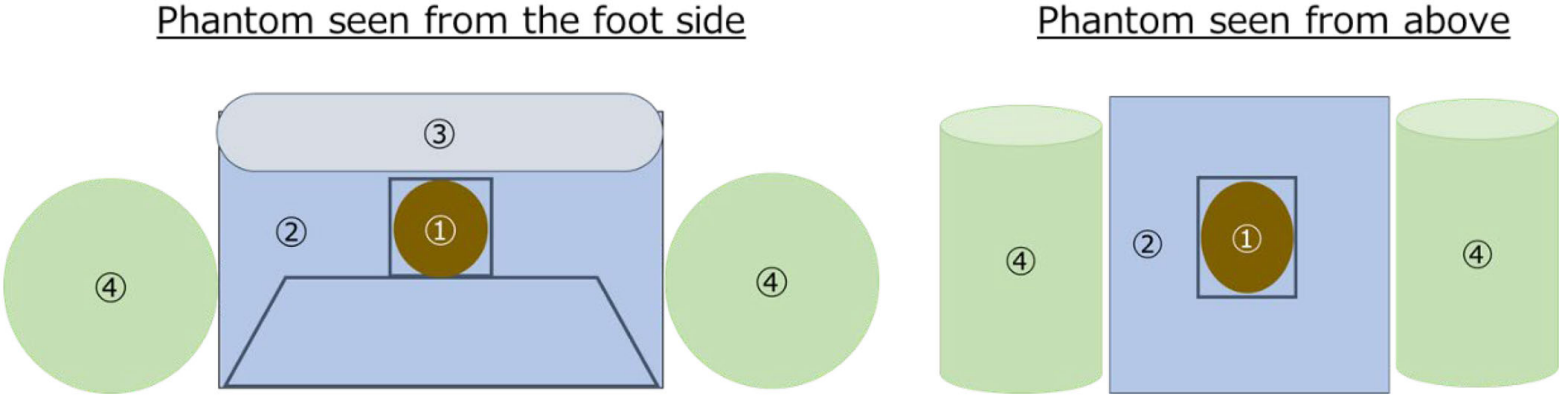
Structure of the prostate‐mimicking phantom based on a kiwifruit. The phantom mimicked human prostate relaxation times (measured values: T1 = 1603.3 ms, T2 = 72.3 ms). The numbers represent: (1) kiwifruit containing an implanted gold fiducial marker; (2) main 7‐liter container filled with pure water; (3) polyethylene bag containing an agar‐water mixture (10 g/L) for stabilization; and (4) cylindrical containers filled with a nickel sulfate solution (Solution N) for signal stability.

A 10.0‑mm‑long GFM (0.5 mm diameter, VISICOIL, IBL, Florida, USA) was inserted into the kiwi fruit. To establish a rigorous ground‐truth for our accuracy assessment, the physical dimensions of the GFM were verified prior to insertion using an analog caliper (Series No. 530–101; Mitutoyo Corp., Kawasaki, Japan). The kiwi was placed at the center of a 7‑liter container (approximately 29 cm × 18 cm × 18 cm) filled with pure water. To provide structural stabilization and create a consistent surrounding environment, a resealable polyethylene bag (27.3 cm × 26.8 cm) containing a mixture of 1 liter of water and 10 g of agar was placed on top of the container. Additionally, two cylindrical containers (11 cm diameter, 20 cm height) were positioned at both ends of the main container.^16^ These containers were filled with a nickel sulfate solution composed of 3.75 g of NiSO_4_·6H_2_O and 5 g of NaCl per 1000 g of distilled water to ensure signal stability and mimic anatomical context (Figure 1).^16^

### 2.2 Image acquisition

Image acquisition was conducted using a 3.0T MRI scanner (Ingenia Elition, Philips Healthcare, the Netherlands) and a 32‐channel body coil. A 3D ME GRE sequence was employed with the following parameters: repetition time of 25 ms; echo times of 4.8 ms, 9.7 ms, 15.0 ms; flip angle of 15°; field of view of 230 × 230 mm; receiver bandwidth of 394.6 Hz/pixel; and one signal average, acquired matrix of 232 × 231. The acquired matrix (232 × 231) was reconstructed to 288 × 288 for display on the treatment planning system (TPS), yielding an interpolated in‐plane resolution of 0.80 × 0.80 mm^2^. Slice thicknesses of 1.0, 2.0, and 3.0 mm were selected to represent standard clinical protocols for prostate radiotherapy planning.^17^ While a 3.0 mm slice thickness is commonly reported as a pragmatic balance between spatial resolution, signal‐to‐noise ratio,^7^, ^10^, ^18^ and scan time, thinner slices (1.0 and 2.0 mm) are increasingly utilized to minimize partial‐volume blurring and improve the precision of fiducial localization in high‐resolution or MRI‐only workflows.^19^, ^20^, ^21^, ^22^ For each slice thickness, the phantom was positioned at 12 different horizontal angles relative to the table (0°, ± 15°, ± 30°, ± 45°, ± 60°, ± 75°, and 90°) and images were acquired (Table 1). This range covers the full geometric spectrum of GFM orientations relative to the static magnetic field (B_0_), accounting for variations in patient anatomy and implantation techniques such as transperineal or transrectal approaches. The angles were set using a full‐circle protractor placed on the container, and the manual setup uncertainty was estimated to be approximately ± 2°–3°. All images were transferred to the TPS (Eclipse Ver. 16.1, Varian Medical Systems).

**TABLE 1:** MRI acquisition parameters for the 3D ME GRE sequence.

Parameter	Value
MRI system	3.0T (Ingenia Elition, Philips Healthcare)
Sequence	3D Multi‐echo Gradient Recalled Echo (ME GRE)
TR / TE (ms)	25 / 4.8, 9.7, 15.0
Flip angle	15°
Field of view (mm)	230 × 230
Receiver bandwidth (Hz/pixel)	394.6
Number of signal averages	1
Acquired matrix	232 × 231
Reconstructed matrix	288 × 288
Interpolated in‐plane resolution (mm)	0.80 × 0.80
Reconstructed Voxel size (mm^3^)	0.80 × 0.80 × (1.0, 2.0, or 3.0)
Slice thickness (mm)	1.0, 2.0, 3.0 (Variables)
Orientation angles	0°, ± 15°, ± 30°, ± 45°, ± 60°, ± 75°, 90° (Variables)

Abbreviations: TE, echo time; TR, repetition time.

### 2.3 Image analysis

Five multidisciplinary observers—one radiation oncologist, two radiologists, and two radiological technologists, all with over five years of experience in radiation therapy—determined the 3D coordinates (*x*, *y, z*) of the GFM tip on the TPS. Each observer performed a single set of measurements for all 36 image conditions. To ensure objectivity, all observers were blinded to the MRI acquisition parameters during the identification process. Each measurement was performed individually at separate workstations or times to prevent communication between observers regarding the coordinate identification results. The coordinate identification was performed using multiplanar reconstruction (MPR), simultaneously viewing axial, sagittal, and coronal planes. To preserve the intrinsic spatial resolution of the acquired data, all measurements were performed on the original voxel grid by disabling slice‑interpolation and image‑smoothing functions within the TPS. This ensured that the identified coordinates reflected the true acquired resolution along the marker's longitudinal axis, while the in‑plane resolution remained interpolated for display purposes. The GFM length (*L*) was calculated using the following Euclidean distance formula (Equation 1):

(1) $$L=\sqrt{{\left(x_{1}-x_{2}\right)}^{2}+{\left(y_{1}-y_{2}\right)}^{2}+{\left(z_{1}-z_{2}\right)}^{2}}$$

where the subscripts 1 and 2 denote the two opposite tips (ends) of the same GFM, and $\left(x_{1},y_{1},z_{1}\right)$ and $\left(x_{2},y_{2},z_{2}\right)$ represent their respective three‐dimensional coordinates as identified by the observers.

**FIGURE 2:**
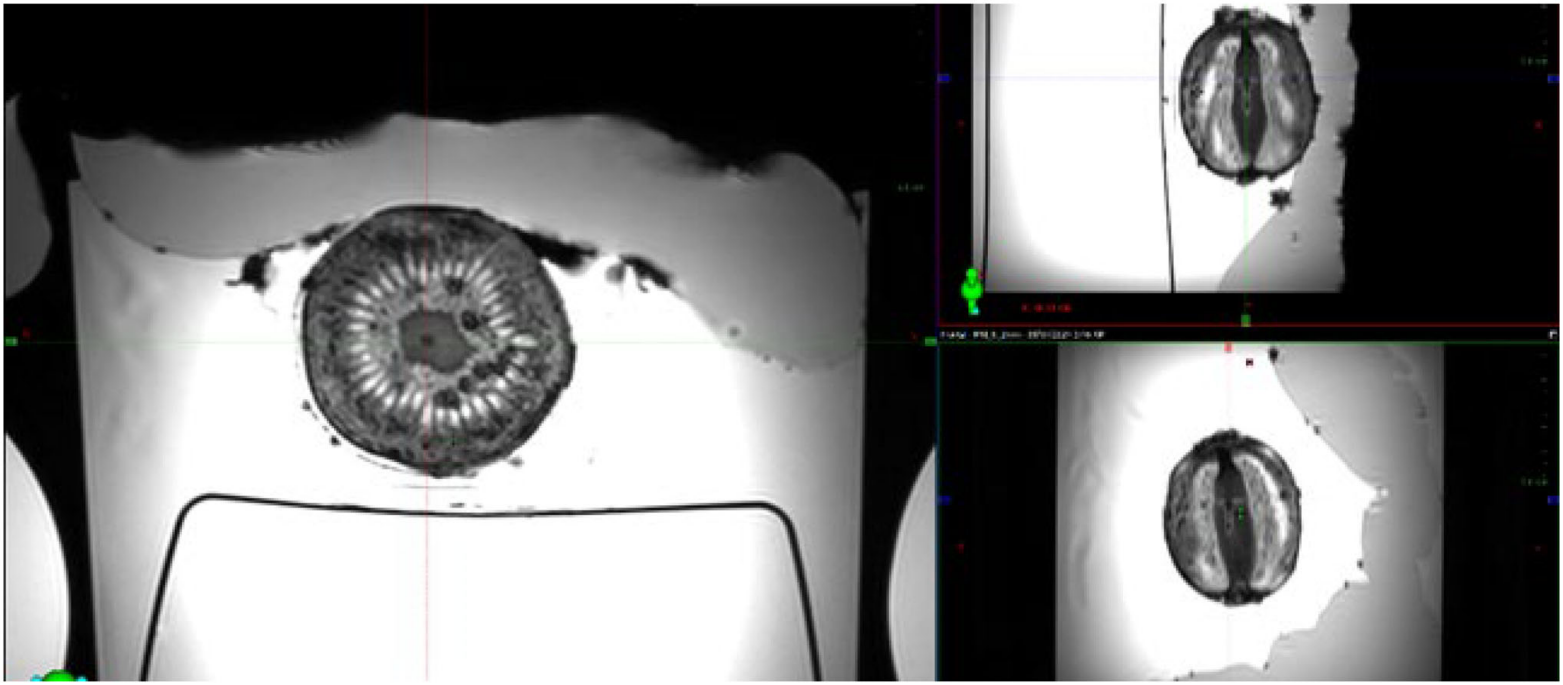
Visualization and coordinate identification of gold fiducial marker (GFM) signal voids on the TPS. The 3D coordinates (*x,y,z*) were defined using the TPS's inherent Cartesian coordinate system, with the marker tips identified at the intersection of the crosshairs in the MPR view. Subjective confidence scores were recorded on a scale of 0 to 100 for each measurement, where 0 represents “complete uncertainty” (boundaries indistinguishable from the background) and 100 represents “absolute certainty” (perfectly defined marker tips with no visual ambiguity).

Confidence scores were recorded with the continuous confidence method (Figure 2). Prior to the measurements, a standardized verbal calibration and consensus training session was conducted to standardize the subjective confidence scoring on a continuous visual analog scale ranging from 0 to 100. The objective of this session was to establish a common baseline among the multidisciplinary observers by explicitly defining anchor points on the scale. A score of 0 was defined as “complete uncertainty”, where the marker boundaries were indistinguishable from the background, making tip identification purely speculative. Conversely, a score of 100 represented “absolute certainty”, where the marker tips were perfectly defined with no visual ambiguity. Based on the consensus reached during the calibration session, a score of 80 or higher was defined as the level of clarity suitable for clinical image matching. Observers were instructed to base their ratings on the perceived clarity of the signal voids and the ease with which the longitudinal edges of the GFM could be distinguished from the surrounding phantom tissue. This process ensured that all observers applied the same criteria regardless of their clinical background.

### 2.4 Statistical analysis

All statistical analyses were performed using SPSS software (version 30, IBM Corp., United States of America). The primary outcome was the GFM measurement error, defined as the difference between the calculated marker length on the TPS and the actual length of 10.0 mm.

The assumptions of normality and homogeneity of variances were evaluated using the Shapiro–Wilk test and Levene's test, respectively. Although these assumptions were partially violated, a two‐way analysis of variance (ANOVA) was applied because the perfectly balanced experimental design (*n* = 15 per cell; *N* = 180 total) provides robustness against moderate deviations from normality and homoscedasticity. When significant main or interaction effects were observed, Tukey's Honestly Significant Difference (HSD) test was used for post‐hoc pairwise comparisons. This test was selected for its ability to strictly control the family‐wise Type I error rate and its established robustness to violations of normality and homoscedasticity in perfectly balanced experimental designs (*n* = 15 per cell).^23^

Inter‑observer reliability among the five observers was assessed using the intraclass correlation coefficient (ICC [2, k]) based on a two‑way mixed‑effects model with absolute agreement. Agreement and potential systematic bias were further examined using Bland–Altman analysis, including the mean difference and 95% limits of agreement (LoA).

To explore the influence of professional background, an independent‑samples t‑test was conducted to compare measurement errors between the physician group (observers A–C) and the radiological technologist group (observers D and E). Pearson's correlation coefficient was used to evaluate the relationship between subjective confidence scores and objective measurement errors. Prior to this analysis, the assumptions for Pearson's correlation, including linearity and homoscedasticity, were verified through visual inspection of scatter plots. Statistical significance was defined as *p* < 0.05.

## 3 RESULTS

### 3.1 Statistical assumptions and inter‐observer reliability

Prior to the main analysis, the assumptions of normality and homogeneity of variances were formally assessed. The Shapiro‐Wilk test (*p* < 0.001) and Levene's test (*p* < 0.001) indicated that the measurement error data were not normally distributed and had unequal variances. These results were formally considered when selecting post‐hoc procedures. However, the two‐way ANOVA and subsequent Tukey HSD tests were maintained because our experimental design was perfectly balanced (*n* = 15 per cell, *N* = 180 total), which provides statistical robustness against violations of these assumptions. Additionally, visual inspection of the scatter plots confirmed that the relationship between confidence scores and measurement errors satisfied the requirements for linear correlation analysis.

**FIGURE 3:**
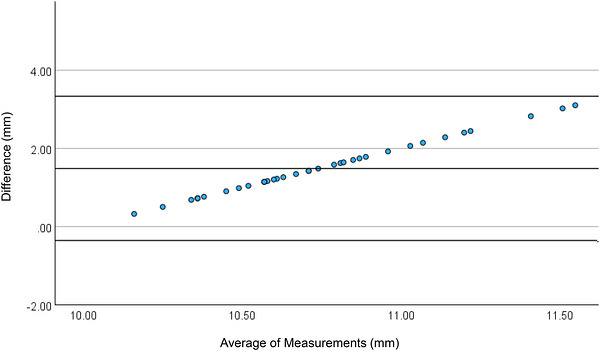
Bland–Altman plot assessing the agreement of gold fiducial marker (GFM) length measurements among the five multidisciplinary observers. The horizontal center line represents the mean bias (1.49 mm), indicating a systematic overestimation of the marker length across all observations. The upper and lower dashed lines represent the 95% limits of agreement (LoA), ranging from −0.36 to 3.33 mm. The plot illustrates high inter‐observer reliability, as evidenced by the intraclass correlation coefficient (ICC = 0.804). A slight proportional bias is observed, where the measurement difference (Diff) tends to increase as the average measured GFM length (Average) increases.

To evaluate inter‐observer reliability among the five observers, the intraclass correlation coefficient (ICC [2, k]) was calculated. The analysis revealed an ICC of  .804 (95% confidence interval [CI]: 0.655–0.894, *p* < 0.001), indicating excellent agreement across multidisciplinary observers. Furthermore, Bland‐Altman analysis (Figure 3) confirmed this reliability, showing a mean bias of 1.49 mm (95% limits of agreement [LoA]: −0.36 to 3.33 mm). A proportional bias was observed, where the measurement difference tended to increase as the average GFM length increased.

### 3.2 Main effects of slice thickness and orientation

The two‐way ANOVA (Table 2) demonstrated that both slice thickness (*F*(2, 144) = 32.03, *p* < 0.001) and horizontal orientation angle (*F*(11, 144) = 4.06, *p* < 0.001) had significant main effects on the GFM measurement error.

**TABLE 2:** Two‐way ANOVA results for comparisons of GFM measurement error.

Source	*df*	Mean square	*F*‐value	*p*‐value
**Slice thickness**	2	16.225	**32.027**	**< 0.001**
**Orientation angle**	11	2.059	**4.064**	**< 0.001**
**Interaction (thickness × angle)**	22	1.376	**2.715**	**< 0.001**
Error	144	0.507	–	–

Abbreviation: df, degrees of freedom.

**FIGURE 4:**
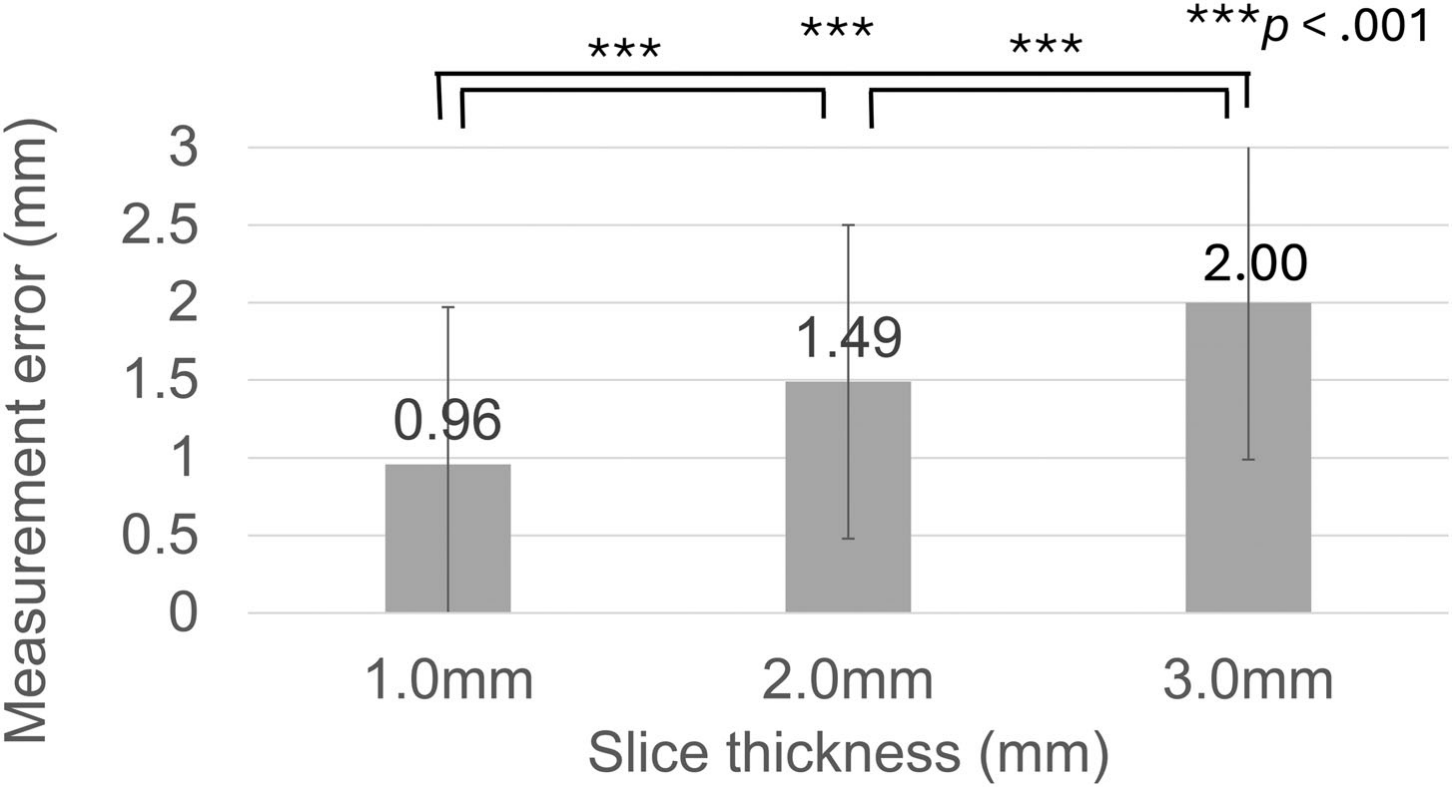
Impact of MRI slice thickness (1.0, 2.0, and 3.0 mm) on mean gold fiducial marker (GFM) measurement error.

Post‐hoc Tukey HSD tests revealed significant differences between all slice thickness pairs (*p* < 0.001). As shown in Figure 4, measurement errors increased significantly with slice thickness: 0.96 mm (95% CI: 0.70–1.22 mm) for 1.0 mm slices, 1.49 mm (95% CI: 1.23–1.75 mm) for 2.0 mm slices, and 2.00 mm (95% CI: 1.74–2.26 mm) for 3.0 mm slices.

### 3.3 Interaction effects and orientation peak errors

A statistically significant interaction between slice thickness and orientation angle was observed (*F*(22, 144) = 2.72, *p* < 0.001), indicating that the impact of slice thickness depends on the marker orientation.

**FIGURE 5:**
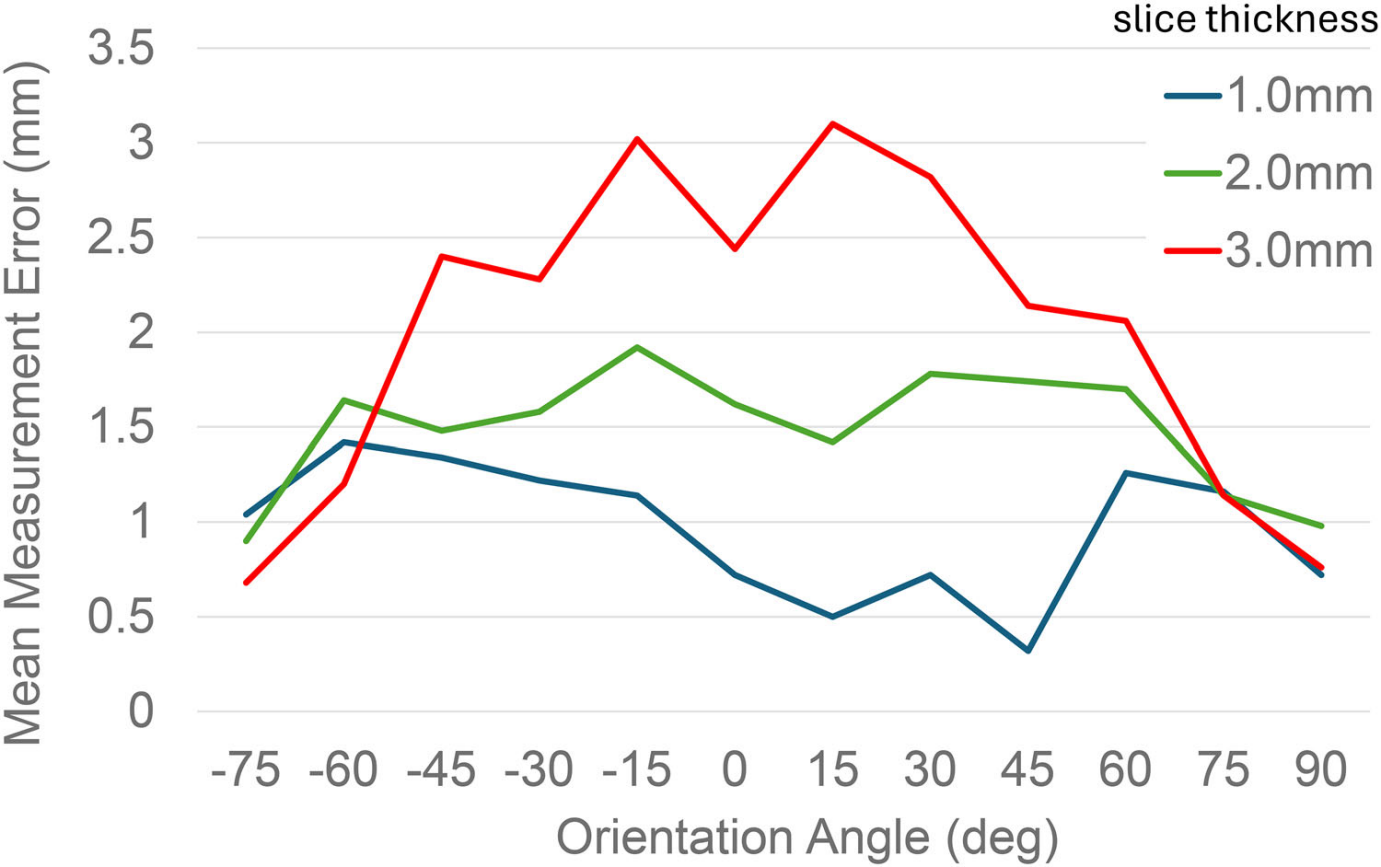
Interaction plot of orientation angle and slice thickness on gold fiducial marker (GFM) measurement error. Two‐way analysis of variance (ANOVA) revealed a statistically significant interaction between slice thickness and orientation angle (*F*(22, 144) = 2.715, *p* < 0.001).

Detailed analysis of the 36 individual combinations (Table 3 and Figure 5) revealed that the measurement error reached its peak at shallow orientation angles. Specifically, using 3.0 mm slices at a 15° orientation resulted in the maximum observed error of 3.10 mm. Similarly, a peak error of 3.02 mm was observed at −15° with 3.0 mm slices. In contrast, at a vertical orientation (90°), measurement errors remained stable and consistently low (0.72–0.76 mm) across all slice thicknesses.

**TABLE 3:** Mean gold fiducial marker (GFM) length measurement errors (mm) for the 36 combinations of MRI slice thickness and horizontal orientation angle.

	Slice thickness		
Orientation angle (deg)	1 mm	2 mm	3 mm
−75	1.040	0.900	0.680
−60	1.420	1.640	1.200
−45	1.340	1.480	2.400
−30	1.220	1.580	2.280
**−15**	1.140	1.920	**3.020**
0 (horizontal)	0.720	1.620	2.440
**15**	0.500	1.420	**3.100** ^*^
30	0.720	1.780	2.820
45	0.320	1.740	2.140
60	1.260	1.700	2.060
75	1.160	1.140	1.140
90 (vertical)	0.720	0.980	0.760

Measurement error is defined as (measured length–actual GFM length of 10.0 mm). Positive values indicate overestimation. Bold values represent errors exceeding 3.0 mm, which are clinically significant.

* indicates the maximum error observed in the study (3.10 mm).

Abbreviations: GFM, gold fiducial marker; MRI, magnetic resonance imaging.

### 3.4 Comparison of measurement errors between professions

To address the impact of professional background, measurement errors were compared between the physician group (observers A, B, and C) and the radiological technologist group (observers D and E). An independent‐samples t‐test revealed a statistically significant difference (*t*(159.94) = −2.48, *p* = 0.014). The physician group had a mean error of 1.35 mm (standard deviation (SD): 0.95), while the technologist group had a mean error of 1.69 mm (SD: 0.89). The mean difference was −0.34 mm (95% CI: −0.62 to −0.07 mm). Despite this statistical significance, the absolute difference of 0.34 mm is clinically negligible compared to the 1.04 mm difference between 1.0 mm and 3.0 mm slice thicknesses.

### 3.5 Subjective confidence assessment

The subjective confidence scores across all 180 measurements reflected the varying degrees of visual ambiguity encountered by the observers. The mean confidence scores decreased as slice thickness increased, with values of 88.3 ± 8.4 for 1.0 mm, 80.4 ± 9.6 for 2.0 mm, and 61.2 ± 13.1 for 3.0 mm slices. Notably, the lowest individual confidence scores were recorded for the 3.0 mm slice at 15°, coinciding with the condition in which the maximum geometric error occurred.

**FIGURE 6:**
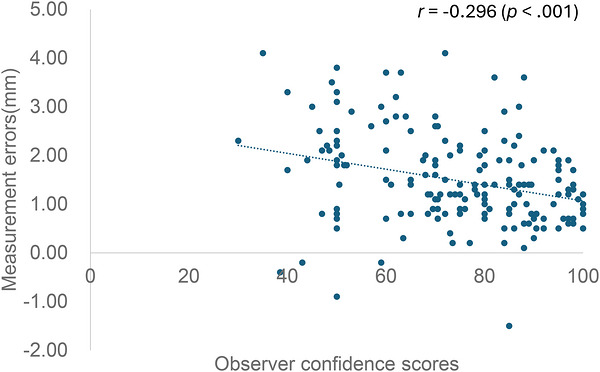
Scatter plot showing the correlation between observer confidence scores and gold fiducial marker (GFM) measurement errors. A significant negative correlation was observed (Pearson's correlation coefficient *r* = −0.296, *p *< 0.001), indicating that lower visualization clarity (lower confidence) is associated with increased physical localization error.

Pearson's correlation analysis revealed a significant negative correlation between observer confidence scores and physical measurement errors (*r *= −0.296, *p* < 0.001) (Figure 6). This result demonstrates that higher visual ambiguity (lower confidence) is statistically associated with greater localization uncertainty.

However, the small magnitude of the correlation coefficient indicates a weak relationship, suggesting that high observer confidence does not necessarily guarantee low geometric error. This result indicates that conditions associated with larger geometric errors, such as thicker slices and specific shallow angles, also resulted in lower subjective certainty during marker identification.

## 4 DISCUSSION

The primary finding of this study is that GFM visualization accuracy in 3D ME GRE sequences is jointly determined by MRI slice thickness and marker orientation angle. We identified a statistically significant interaction between these two factors (*p* < 0.001), demonstrating that while localization remains accurate under optimal conditions, measurement error can reach a peak value of 3.10 mm—representing a 31% overestimation of the marker's 10.0 mm physical length—specifically under the worst‐case combination of 3.0 mm slices and a shallow 15° orientation. It is important to note that this peak error is substantially higher than the mean error of 0.96 mm observed with 1.0 mm slices, which maintained sub‐millimeter accuracy regardless of orientation. Unlike previous investigations that primarily focused on comparing marker visibility across conventional sequences to determine which protocol is most effective for identifying markers as signal voids,^9^, ^10^ this research provides a novel quantitative assessment of geometric uncertainty. By shifting the focus from subjective visibility to physical measurement accuracy, this study offers a technical foundation for multidisciplinary treatment planning that was not fully elucidated in earlier Phantom studies.

To ensure the robustness of our results, we evaluated inter‐observer reliability among five multidisciplinary experts. The ICC of  .804 (95% CI: 0.655–0.894, *p *< 0.001) indicated excellent agreement across observers. Furthermore, Bland‐Altman analysis (Figure 3) confirmed a systematic overestimation bias of 1.49 mm. We compared measurement errors between the physician group and the radiological technologist group. While a statistically significant difference was found (*p* = 0.014), the absolute difference was only 0.34 mm. From a clinical perspective, this variation is negligible compared to the impact of slice thickness (approximately a 1.0 mm difference between 1.0 and 3.0 mm slices). This finding underscores the robustness of the 1.0 mm slice protocol. Furthermore, we consider the multidisciplinary nature of our observer group to be a technical strength of this study; the high inter‐observer reliability (ICC = 0.804) among experts from diverse clinical backgrounds suggests that the identified geometric uncertainties are inherent to the MRI acquisition parameters themselves and are reproducible across a typical radiation oncology team.

Increased slice thickness is a major contributor to measurement error; the mean error increased significantly from 0.96 mm with a 1.0 mm slice thickness to 2.00 mm with a 3.0 mm slice thickness. Physically, this is due to the partial‐volume effect (PVE), a well‐documented phenomenon in MRI where multiple tissue properties or magnetic field inhomogeneities within a single voxel are averaged.^11^, ^24^ As slice thickness increases, PVE amplifies volumetric and shape errors, leading to a significant degradation of geometric accuracy in marker localization.^24^ In the context of GFM, the magnetic field gradients at the marker‐tissue interface are averaged across the slice direction, resulting in an elongated signal loss that obscures the true position of the marker tips. These results suggest that acquiring thin‐slice images (1.0 mm) is highly desirable to minimize these PVE‐induced uncertainties and maintain sub‐millimeter localization accuracy. Furthermore, as noted in clinical practice, while the physical orientation of an implanted GFM is often a factor that clinicians can do very little to control after implantation, MRI slice thickness is a controllable acquisition parameter. Given that clinicians have limited intervention over the marker angle, adopting a 1.0 mm slice thickness protocol serves as a critical technical safeguard to ensure consistent sub‐millimeter localization accuracy regardless of the marker's physical orientation. This is particularly important in clinical cases where high localization accuracy is essential for treatment quality and safety.

A key finding of this study is the orientation dependence, with measurement errors peaking at shallow tilt angles near ± 15°, which can be explained by the spatial distribution of the susceptibility‑induced magnetic field perturbation (ΔB) around the GFM. According to the dipole model, the local field shift generated by a cylindrical metallic object is proportional to (1 ‐ 3cos^2^ *θ*), where *θ* is the angle between the marker's long axis and the B_0_.^11^

These local gradients produce intra‑voxel dephasing and signal voids, known as the “blooming effect.^11^” Although the total void volume is generally largest when the object is perpendicular to B_0_, our results show that linear measurement error is most pronounced at shallow angles.^25^

This behavior in the 3.0‑mm slices is primarily due to a longitudinal accumulation effect, in which susceptibility‑induced signal voids overlap across multiple voxels along the marker's long axis within the thick slice, blurring the marker boundaries and leading to overestimation of its length.^25^ In contrast, the 1.0‑mm slice data did not exhibit a peak at ± 15° but instead showed larger errors at higher angles (approximately 60°) and slight asymmetry between positive and negative orientations. The thinner slice thickness reduces longitudinal accumulation and confines susceptibility artifacts more effectively, diminishing the overestimation mechanism observed in the 3.0‑mm slices. Under these higher‑resolution conditions, smaller effects—such as manual phantom‑orientation uncertainty (± 2–3°) and image noise—may exert a relatively stronger influence, contributing to the shifted peak angle and subtle asymmetry.

At a vertical orientation (90°), the marker's long axis is parallel to the slice‑selection gradient, minimizing longitudinal artifact spread and yielding stable low errors (0.72–0.76 mm) regardless of slice thickness. However, this stability in length measurement does not imply that slice resolution has minimal impact on true 3D localization accuracy, because the error metric used here reflects only the difference in measured length rather than coordinate deviation relative to a reference.

To avoid confusion, it should be noted that while slice thickness represents the acquired (non‐interpolated) resolution, the in‐plane resolution displayed on the TPS is interpolated, and therefore these two dimensions reflect different aspects of spatial resolution.

The significant negative correlation observed between observer confidence and measurement error (*r* = −0.296, *p* < 0.001) objectively confirms that visual ambiguity is linked to coordinate identification uncertainty. However, while statistically significant, the correlation coefficient is relatively weak, emphasizing that statistical significance does not inherently imply strong predictive value. Therefore, subjective confidence should be interpreted cautiously as a qualitative indicator of visual ambiguity rather than a definitive predictor or quantitative surrogate of absolute geometric accuracy. Nonetheless, conditions that make identification difficult for clinicians often coincide with larger physical distortions, providing a practical warning sign during the registration workflow.

From a clinical standpoint, a systematic error exceeding 3 mm poses a risk to the precision of radiotherapy. To illustrate this impact, we performed a theoretical simulation using Van Herk's margin recipe (*M* = 2.5Σ + 0.7*σ*), assuming that the observed peak measurement error (3.10 mm) represents a constant systematic shift (Σ) within a specific imaging protocol. This simulation suggests that such an error could theoretically necessitate a margin expansion of over 7 mm. However, this application has inherent limitations: Van Herk's framework is designed for population‐level stochastic uncertainties and assumes that all error components are independent and Gaussian. In clinical practice, the total systematic error (Σ) is a combination of multiple factors, including organ motion and setup variations, which were not captured in our static phantom model. Therefore, our calculation should be interpreted not as a definitive clinical mandate, but as a simplified physical baseline demonstrating how a single imaging‐related uncertainty can disproportionately consume the available PTV margin (typically 5–10 mm) and increase the risk of a ‘geometric miss’.

These findings, interpreted as a theoretical risk simulation rather than a direct clinical mandate, underscore the importance of minimizing inherent registration uncertainties as recommended by AAPM Task Group 132.^3^

This study has several limitations that must be acknowledged. First, as a static phantom experiment, this study does not account for dynamic patient motion effects (including respiratory and physiological motion) or the inherent complexities of deformable anatomy. In clinical scenarios, the prostate is subject to non‐rigid deformations caused by variations in rectal and bladder filling, alongside susceptibility effects from rectal gas, which were not captured in this model. Consequently, the geometric uncertainties identified here represent a controlled physical baseline that may be further exacerbated by these unpredictable clinical variables. Second, only one type and size of GFM was evaluated. Since susceptibility artifacts depend strongly on marker composition and geometry, these findings may not be directly generalizable to all commercially available markers, such as solid cylindrical markers or those with different dimensions, which may exhibit varying degrees of the blooming effect. Third, the coordinate identification was performed manually, which, despite the high ICC, retains an inherent level of subjectivity compared to automated algorithms. Additionally, intra‐observer variability was not assessed as measurements were not repeated by the same individuals. However, the excellent inter‐observer agreement (ICC = 0.804) among five experts from different clinical backgrounds suggests that the measurement protocol is highly reproducible. Another limitation is the weak correlation between subjective confidence and objective error (*r* = −0.296), which prevents the use of observer certainty as a reliable quantitative surrogate for measurement precision. However, from a practical perspective, low confidence can still serve as a qualitative warning sign of imaging conditions prone to higher geometric uncertainty. Finally, while we illustrated the potential impact on PTV margins using a theoretical formula, this was a simplified simulation based on a static environment. In clinical practice, the necessary margins are influenced by complex interactions of multiple stochastic factors that were not captured in this phantom model. Future clinical validation using in vivo patient data and comparative analysis with advanced sequences like susceptibility‐weighted imaging (SWI) are necessary to confirm these findings in a realistic clinical environment.

## 5 CONCLUSION

In conclusion, this study demonstrates that MRI slice thickness and marker orientation jointly determine the geometric accuracy of GFM visualization in 3D ME GRE sequences. The findings highlight a critical geometric risk associated with standard 3.0 mm slice protocols, where specific orientation angles can lead to significant overestimation of marker dimensions. In contrast, our results show that consistent sub‐millimeter localization accuracy can be maintained across all orientations by adopting a 1.0 mm slice thickness protocol. While clinicians must balance spatial resolution with practical considerations such as scan time and signal‐to‐noise ratio, implementing high‐resolution thin‐slice imaging is highly desirable to ensure the quality and safety of high‐precision prostate radiotherapy planning.

Furthermore, the significant negative correlation between observer confidence and measurement error suggests that thicker slices create visual ambiguity, thereby increasing localization uncertainty. To achieve sub‐millimeter localization accuracy, our findings suggest that a 1.0 mm slice thickness protocol is highly desirable for ME GRE sequences. While clinical implementation requires balancing spatial resolution with practical factors such as scan time, such a protocol ensures consistent geometric precision regardless of marker orientation, thereby enhancing the quality and safety of radiotherapy.

## AUTHOR CONTRIBUTIONS

Conceptualization of study (Aika Hayashi, Yasukata Takahashi, Naoko Sanuki), Execution of study (Aika Hayashi, Yasufumi Yamashita, Yoshitaka Suzuki), Data curation and analysis (Aika Hayashi, Hiroe Muto), Software (Aika Hayashi, Hiroe Muto, Kanae Matsuura), Writing (Aika Hayashi, Hiroe Muto), Review (Aika Hayashi, Hiroe Muto, Kanae Matsuura).

## CONFLICT OF INTEREST STATEMENT

The authors declare no conflicts of interest.

## Data Availability

The data that support the findings of this study are available from the corresponding author upon reasonable request.
